# Birth mode-dependent association between pre-pregnancy maternal weight status and the neonatal intestinal microbiome

**DOI:** 10.1038/srep23133

**Published:** 2016-04-01

**Authors:** Noel T. Mueller, Hakdong Shin, Aline Pizoni, Isabel C. Werlang, Ursula Matte, Marcelo Z. Goldani, Helena A. S. Goldani, Maria Gloria Dominguez-Bello

**Affiliations:** 1 Department of Epidemiology, Johns Hopkins Bloomberg School of Public Health, Baltimore, MD, USA; 2 New York University School of Medicine, New York, NY, USA; 3 Post-Graduate Program Sciences in Gastroenterology and Hepatology, Faculty of Medicine, Federal University of Rio Grande do Sul, Porto Alegre – RS, Brazil; 4 Laboratory of Translational Pediatrics, Hospital de Clínicas de Porto Alegre, Federal University of Rio Grande do Sul, Porto Alegre – RS, Brazil; 5 Department of Genetics, Federal University of Rio Grande do Sul, Porto Alegre – RS, Brazil; 6 Department of Pediatrics, Faculty of Medicine, Federal University of Rio Grande do Sul, Porto Alegre – RS, Brazil

## Abstract

The intestinal microbiome is a unique ecosystem that influences metabolism in humans. Experimental evidence indicates that intestinal microbiota can transfer an obese phenotype from humans to mice. Since mothers transmit intestinal microbiota to their offspring during labor, we hypothesized that among vaginal deliveries, maternal body mass index is associated with neonatal gut microbiota composition. We report the association of maternal pre-pregnancy body mass index on stool microbiota from 74 neonates, 18 born vaginally (5 to overweight or obese mothers) and 56 by elective C-section (26 to overweight or obese mothers). Compared to neonates delivered vaginally to normal weight mothers, neonates born to overweight or obese mothers had a distinct gut microbiota community structure (weighted UniFrac distance PERMANOVA, *p* < 0.001), enriched in *Bacteroides* and depleted in *Enterococcus, Acinetobacter, Pseudomonas*, and *Hydrogenophilus*. We show that these microbial signatures are predicted to result in functional differences in metabolic signaling and energy regulation. In contrast, among elective Cesarean deliveries, maternal body mass index was not associated with neonatal gut microbiota community structure (weighted UniFrac distance PERMANOVA, *p* = 0.628). Our findings indicate that excess maternal pre-pregnancy weight is associated with differences in neonatal acquisition of microbiota during vaginal delivery, but not Cesarean delivery. These differences may translate to altered maintenance of metabolic health in the offspring.

An increasing number of pregnant women around the world are overweight or obese^1^,^2^,^3^. Maternal overweight and obesity may result in 2 to 5 fold greater risk of obesity for their children^4^,^5^,^6^. Breaking this vicious cycle of obesity requires a better understanding of its underpinnings. The association between excess maternal weight and childhood obesity is not fully explained by known genetic polymorphisms or lifestyle factors shared between the mother and her offspring^7^. A novel hypothesis is that at least part of the intergenerational obesity association is due to mother-to-newborn transmission of obesogenic microbiota^8^,^9^.

As women progress through pregnancy their intestinal microbiota undergo marked shifts to promote weight gain and accommodate the growth of the fetus^10^. Normal pregnancy-related remodeling of the intestinal microbiota is modified by the amount of weight with which women enter pregnancy^11^. Specifically, compared to their normal weight counterparts, women carrying excess pre-pregnancy weight have been shown to have higher levels of *Bacteroides* in the third trimester^11^. Considering that the mother is the primary source of microbiota for the newborn^9^, and that transmission of obese human microbiota to germ-free mice alters the assembly of their intestinal microbiome in a manner that favors adipogenesis^12^,^13^,^14^, it is plausible that weight-related modifications to the maternal intestinal microbiome during pregnancy may impact assembly of the infant intestinal microbiome and, in turn, host metabolism.

Indeed, previous studies have shown that maternal pre-pregnancy body mass index (BMI) is associated with the intestinal microbial community structure of infants’ stool at 1 month, 6 months^15^, and 2 years of age^16^. But it is still unknown whether the structuring of the infant intestinal microbiome by maternal weight status is a result of mother-to-offspring transmission of microbiota during labor, after birth, or even prior to birth, as has been suggested by recent study^17^. In our study, we examine the role of maternal pre-pregnancy BMI in the assembly of the intestinal microbiome of neonates delivered vaginally vs. by elective Cesarean section (CS) without exposure to membrane rupture. We find that maternal pre-pregnancy BMI is associated with microbial community structure and taxomonic differences in neonatal stool if the neonate is delivered vaginally, but not if they are delivered by CS. Moreover, we show that these microbial alterations are predicted to translate into functional metagenomic differences in metabolism and energy regulation.

## Results

### Study population

The mean ± SD maternal pre-pregnancy BMI from our analytic sample was 24.7 ± 4.5 kg/m^2^, and for only the overweight or obese group it was 28.8 ± 3.7 kg/m^2^. The hospital from which the participants were recruited is a private hospital with high socioeconomic status patients. Almost all of the women (70 of 74) in our sample described themselves as “white” (or “branco” in Portuguese). Other clinical characteristics of the study sample are presented in Table 1.

### Characterization of neonatal intestinal microbiome

To investigate the contribution of maternal body weight to the establishment of the instestinal microbiome of newborns, we undertook 16S ribosomal RNA (rRNA) sequencing on the second day stool of 56 elective CS neonates not exposed to ruptured membranes (26 from overweight or obese mothers) and 21 vaginally delivered (VD) neonates (5 from overweight or obese mothers). After quality filtering (pair-end, Phred > =Q20), we obtained 725,089 sequences from 74 neonatal stool samples; on average ± SEM, 9,799 ± 474 reads per sample (Table S1), binned to 3,717 type of OTUs. All four major bacterial phyla (*Actinobacteria, Bacteroidetes, Firmicutes*, and *Proteobacteria*) were represented in the neonatal stool, accounting for ~99% of the microbial content in each sample.

### Delivery mode, maternal body mass index, and neonatal intestinal microbiome

The microbial beta diversity of stool (weighted and unweighted UniFrac distance PERMANOVA, *p* < 0.001) was different between neonates born via CS vs. VD (Figure S1), but there were no statistical differences in measures of alpha diversity, namely phylogenetic diversity (*p* = 0.07), number of observed species (*p* = 0.29) or species richness (*p* = 0.25) (Figure S2).

**Figure 1:**
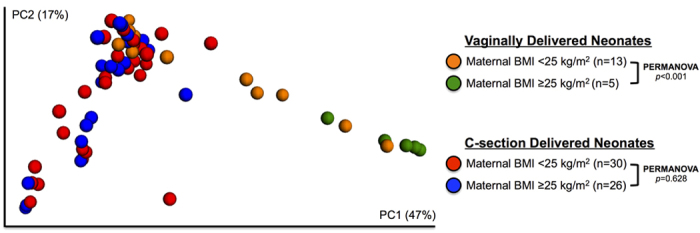
Bacterial beta diversity in neonatal feces according to mode of delivery and maternal pre-pregnancy body mass index (BMI). Weighted UniFrac distances were used to evaluate beta diversity. PERMANOVA was used to test dissimilarity.

We then stratified by delivery mode (CS vs. VD) and compared the microbial communities in stool from neonates born to normal weight vs. overweight or obese (OWOB) mothers in each stratum. In VD neonates’ stool there were statistically significant differences in microbial beta diversity between those born to OWOB vs. normal weight mothers (weighted UniFrac distance PERMANOVA, *p* < 0.001). Consistent with the PERMANOVA results, the microbial communities in the stool of vaginally delivered neonates showed some separation according to maternal pre-pregnancy BMI in unweighted UniFrac principal coordinate analysis (PCoA) plots (Fig. 1). In contrast, maternal pre-pregnancy BMI was not associated with microbial beta diversity of stool from CS delivered neonates (weighted UniFrac distance PERMANOVA, *p* = 0.628). There was no evidence from PCoA that microbial communities in stools from CS-delivered neonates separated according to maternal pre-pregnancy BMI (Fig. 1). As illustrated in Figure S3, there were no differences in measures of alpha diversity, including phylogenetic diversity, number of observed species, or species richness (all *p* values > 0.6) between neonates born to normal weight vs. OWOB mothers in either strata of delivery mode.

**Figure 2:**
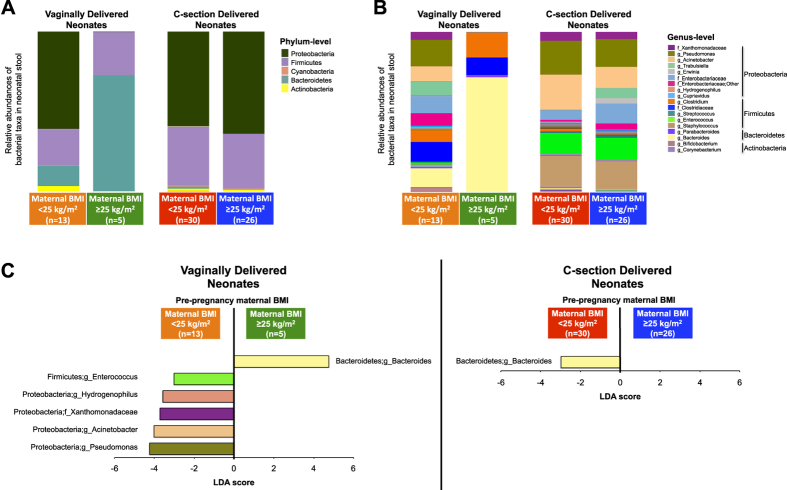
Differences in relative abundance of bacterial taxa in neonatal feces, jointly stratified by delivery mode and maternal pre-pregnancy BMI. (**A,B**) Bacterial taxa plots at the phylum (**A**) and genus (**B**) levels. Each taxa >1% of the average relative abundance in groups is indicated by a different color. Taxa are reported at the lowest identifiable level, indicated by the letter preceding the underscore: f, family; g, genus. **C.** Histogram of biomarker bacteria in each group. Linear Discriminant Analysis (LDA) Effect Size (>3.0-fold) was used to determine statistically significant biomarkers.

Next we compared the relative abundances of microbial taxa [using bacterial taxa plots and Linear Discriminant Analysis (LDA) Effect Size (LEfSe); LDA > 3.0 fold considered significant] in the stools of CS vs. VD neonates (Figure S4) and the stools of neonates born to OWOB vs. normal weight mothers, according to delivery mode strata (Figure 2 and Figure S5). Among the VD group, compared to neonates born to normal weight mothers, neonates born to OWOB mothers had stool enriched in the bacterial genus *Bacteroides* (primarily driven by *B. vulgatus*, followed by *B. dorei, B. caccae, B. xylanisolvens, B. stercoris*, and *B. fragilis* species; Table S2), but depleted in the family *Xanthomonadaceae*, and depleted in the genera *Pseudomonas, Acinetobacter, Hydrogenophilus, Enterococcus*, (Fig. 2; LDA > 3.0). Pre-pregnancy maternal BMI was not associated with taxonomic differences in the stool of CS neonates, except one minor difference in the *Bacteroides* genus (driven by *B. vulgatus*; Table S2) which, contrary to the VD group, was higher in neonates born to normal weight mothers (Fig. 2; LDA > 3.0), but at much lower abundance (relative abundance in normal weight group = 1.0% vs. OWOB group = 0.1%).

We then used PICRUSt predictive functional profiling^18^ to examine if pre-pregnancy BMI was associated with altered metabolic functioning of the microbial groups (Table 2). Compared to neonates born vaginally to normal weight mothers, neonates born vaginally to OWOB mothers had higher bacterial gene content related to metabolism of several carbohydrates (fructose, mannose, galactose, and pentose). Pathways related to fatty acid metabolism were overrepresented in VD neonates born to normal weight mothers (Table 2; all comparisons use an LDA > 3.0). In contrast, but consistent with 16S rRNA results, predictive funtional profiling of stool from CS-delivered neonates showed no differences by pre-pregnancy maternal BMI (Table 2; LDA > 3.0). Results from functional profiling using PICRUSt should be interpreted conservatively because of the known intra-species variation in gene content^18^.

In additional analyses we examined whether the observed associations may be related to differences in prenatal antibiotics or breastfeeding. In the CS group, neither maternal use of antibiotics [taken in the first or second trimesters (n = 11) vs. no use (n = 45)] or breastfeeding [in first 24 hours after birth (n = 49) vs. not (n = 7)] were associated with neonatal fecal microbiota beta diversity (both PERMANOVA *p* values > 0.05; Figure S6). In the VD group, only 3 mothers used antibiotics in pregnancy, and all mothers breastfed in the first 24 hours, so comparisons were not possible.

## Discussion

In this study, we first confirmed previous reports that C-section is the primary perturbation to the natural assembly of neonatal intestinal microbiome shortly after birth^19^,^20^,^21^. We then demonstrated, in analyses stratified by delivery mode, that excess maternal weight was associated with neonatal microbial community structure among neonates delivered vaginally, but not in those delivered by planned C-section without membrane rupture. Herein this study we show for the first time that the association between maternal pre-pregnancy body weight and infant gut microbiome^15^,^16^ may stem from vertical transmission of microbiota during labor and *not* before birth, as was recently suggested^17^.

Among vaginally delivered neonates, the most striking difference between those born to overweight or obese mothers vs. normal weight mothers was the difference in relative abundance of gram-negative *Bacteroides* spp. A Finnish study demonstrated overweight mothers had higher levels of *Bacteroides* genus in the third trimester of pregnancy^11^. Our findings extend these findings, suggesting that the higher levels of *Bacteroides* among overweight women in pregnancy may be vertically transmitted to the newborn during vaginal delivery.

Evidence from previous studies suggests that the association between maternal pre-pregnancy BMI and neonatal microbiome observed in our study may change over time. Collado *et al*. found that maternal pre-pregnacy BMI was negatively associated with fecal levels of *Bacteroides* when the infants from the mothers in the Finnish study were 1 month of age, and pre-pregnancy BMI was not associated with *Bacteroides* when the infants were 6 months of age^15^. In a separate study, Galley *et al*.^16^ demonstrated that, among children from high socioeconomic status families, maternal pre-pregnancy BMI was associated with measures of infant intestinal microbial community structure (beta diversity) and species diversity (alpha diversity) at 1 month, 6 months, and 2 years of age, but it was not associated with levels of *Bacteroides*.

There are several caveats that make it difficult to compare the findings from these studies to ours. First, the studies by Collado *et al*. and Galley *et al*. did not stratify by delivery mode. Our study (Figure S4) and others^19^,^20^,^21^,^22^,^23^,^24^ indicate that colonization of the intestinal tract by *Bacteroides* is delayed in C-section delivered offspring. As overweight mothers are more likely to deliver by C-section, not stratifying by delivery mode may make it appear that overweight mothers deliver infants with lower levels of *Bacteroides*. Another major difference across studies is the timing of stool specimen collection. Previous studies collected stool from 1 month onward whereas we collected at 2 days postpartum. The infant microbiota are highly variable during the first months of life^25^,^26^, and strongly influenced by breastfeeding^20^,^27^, which itself varies by maternal BMI^28^. In our study, all mothers who delivered vaginally initiated breastfeeding within the first 24 hours. Differences in microbiota measurement technique (FCM-FISH and qPCR^15^ *vs.* 454 sequencing^16^ *vs.* Illumina MiSeq) also makes direct comparison between previous studies and ours challenging^29^. Finally, it is important to note that maternal overweight is influenced by a heterogenous mix of genetic and environmental factors, each of which likely has its own effect on microbial community structure. For example, individuals who become overweight or obese consuming a high-fat, high-protein animal-based diet may have high relative abundance of bile-tolerant *Bacteroides* species and low relative abundance of *Firmicutes* species that metabolize plant polysaccharides^30^,^31^,^32^.

The biomarker discovery tool LEfSe also showed lower relative abundance of *Enterococcus, Acinetobacter, Pseudomonas*, and *Hydrogenophilus* in the stool of neonates delivered vaginally to overweight mothers compared to the stool of neonates delivered vaginally to normal weight mothers. *Enterococcus faecalis* FK-23, isolated from the feces of a healthy human subject, has been shown to inhibit development of obesity and hepatic steatosis in mice fed a high fat diet^33^. Double blind, placebo controlled feeding trials have shown that *Enterococcus* subgroups can be increased through a whole grain, prebiotic diet^34^,^35^. *Acinetobacter*, a genus of Gram-negative bacteria, is an antibiotic resistant infectious pathogen found in hospitals^36^. While this genus has been found to be positively associated with obesity in a case-control study of Taiwanese adults, the overall relative abundance in this study was low in cases and controls^37^. The other genera discovered by LEfSe, *Pseudomonas* and *Hydrogenophilus*, have not, to the best of our knowledge, been previously associated with overweight or obesity.

Many of the *Bacteroides* species *(e.g., B. vulgatus/B. dorei, B. caccae, B. xylanisolvens, B. stercoris*, and *B. fragilis*) driving the association between maternal BMI and neonatal gut micobiota in our study are commonly classified in the *B. fragilis* group^38^. Backhed *et al*. showed that germ-free mice incoculated with *B. fragilis* gained excess weight^13^. In prospective cohort studies, colonization with *B. fragilis* at 1 month of age was positively associated with BMI z-score in children following a low-fiber diet at age 4, but inversely associated with BMI z-score in children following a high-fiber diet^39^. Vael *et al*. found a positive association between *B. fragilis* in the feces of 3- and 26-week old infants and BMI z-score at preschool (n = 138)^40^. In contrast, White *et al*. demonstrated, that presence of *Bacteroides* in 30-day-old infants was associated with lower body weight z-scores at 6 months of age in males (n = 108), but not females^41^. Collectivley, previous studies indicate that the association of early-life *Bacteroides* colonization with obesity risk may depend on interactions with other factors.

Through predictive funtional profiling we found that the stool of neonates vaginally delivered by overweight or obese mothers had higher bacterial gene content related to metabolism of several sugar constituents (fructose, mannose, galactose, pentose and glucoronate), amino acids (alanine, aspartate, glutamate and histidine), methane, glycans, sphingolipids and purine, but lower gene content related to the metabolism of other amino acids (lysine, tryptophan, valine, leucine and isoleucine), fatty acids, butanoate, and benzenoate compared to the stool of neonates vaginally delivered to normal weight mothers. There were also differences in bacterial gene content related to enzyme familes and biosynthesis of other secondary metabolites (see Table 2). The enrichment of gene content related to butanoate (i.e., butyrate) metabolism in neonates born to normal weight mothers is particularly intriguing as butyrate is believed to suppress colonic inflammation related to obesity^42^. Functional differences in the metabolism of certain amino acids^43^ methane^44^, sphingolipids^45^, and purine^46^ have also been associated with obesity. Interrogation of these predicted functional differences using true metagenomic shotgun sequencing is warranted in future studies.

The predicted functional differences in carbohydrate metabolism, including the various sugar metabolism pathways enriched in the fecal bacterial DNA of neonates born to overweight or obese women, may be most important for the development of obesity. These differences are likely attributed to the bacterial gene content from *Bacteroides* spp., which have a wide capacity to use diverse types of polysaccharides^47^. All *Bacteroides* spp. can utilize frutose, sucrose, and fructo-oligosaccharides as energy^48^, and they are adept at using oligosaccharides including those found in human breast milk^27^. *Bacteroides* spp., except for *B. vulgatus*, are also able to grow efficiently using one of the long-chain fructans, inulin or levan^48^. *B. caccae* and *B. fragilis* can use inulin with efficiency similar to their use of glucose^48^. Excess of these microbiota may therefore confer more efficient extraction of energy from these non-digestable dietary components and in turn lead to excess weight gain. While it is still unclear how each of the predicted functional differences observed in our study relate to the future health and disease of these neonates, these findings do provide preliminary insight into how metabolic pathways are altered in the first days of life by delivery mode and maternal weight status, and they provide basis for future study.

There are several limitations to our study worth noting. Because we relied upon 16S rRNA sequencing data, and the mothers in our study were not sampled at delivery, we were unable to establish a direct link between the maternal fecal bacteria and the bacterial communities of the newborns’ gastrointestinal tract. However, a recent study, which sampled feces of both mother and newborn, showed that vaginally delivered newborns’ fecal microbiota resembled the fecal microbiota of their mothers^49^. An alternative explanation for our findings is that differences in passive immunity or breast milk oligosaccharide composition, which might select for or transfer certain bacteria to the newborn, influenced the observed association of maternal pre-pregnancy BMI with the neonatal gut microbiome. However, this alternative explanation seems unlikely, as we provide evidence that differences in neonatal microbiome by maternal weight status are confined to vaginally delivered neonates. Furthermore, we demonstrate that breastfeeding initiation within the first 24 hours was not associated with neonatal stool microbiota in our study (Figure S6), indicating that breast milk composition was not a strong determinant of the neonatal microbiome in our sample of neonates. Other limitations to our study are the lack of follow-up measures and the small number of neonates delivered vaginally to overweight or obese mothers. As such, we cannot make inferences about whether the associations extend beyond two days after birth, nor can we rule out the possibility that the associations based on the small sample of neonates delivered vaginally to overweight or obese moms (n = 5) were the result of chance.

## Conclusion

In summary, the observations from our study provide pioneering evidence that maternal weight status is associated with neonatal gut microbiome assemblage differently according to the mode of delivery. Our findings lay the foundation for molecular forensic studies to test the hypothesis that maternal strains of bacteria influenced by her body weight status are transferred to her newborn during labor. Furthermore, large prospective cohort studies are needed to examine whether the differences in the newborn gut microbiota observed in our study persist beyond the first days of life and are associated with infant and childhood weight, metabolism and health outcomes.

## Methods

### Design and subjects

The methods of this study were carried out in accordance with the approved guidelines. The study was approved by the Research and Ethics Committee of the Hospital de Clinicas (protocol no. 11/0388) and the Hospital Mae de Deus (protocol no. 524/11) located in Porto Alegre, Brazil. Subjects for this study were recruited by trained study personnel during 2011. Written informed consent was obtained from mothers of the newborns. As part of the consent process, study personnel discussed with participants the potential risks of participation, including the risks associated with specimen collection, and the possibility that protected health information or de-identified project data stored in a public repository could be accidentally released. The protocol and consent form described precautions taken to reduce these risks. If a participant withdrew consent after providing specimens, remaining specimens and extracted nucleic acids were destroyed.

Women scheduled for either vaginal or elective C-section delivery in a private hospital in Porto Alegre, were invited to participate if they were delivering between 38 and 42 weeks (confirmed by ultrasonography that had been performed before the 20^th^ week of gestational age); did not have HIV/AIDS, preeclampsia, other chronic metabolic diseases (e.g., diabetes, hypertension, or an autoimmune disorder); did not smoke; and were not on a restrictive diet. We excluded women who were administered oral antibiotics in 3^rd^ trimester. Cephalosporin was the perioperative antibiotic predominantly administered for planned Cesarean deliveries in Brazil during our study. Gravidae also had to agree to provide the meconium and first stool from their newborn and to complete a standardized postnatal questionnaire adapted for this study^50^. For vaginal births, only women whose water broke <12 hours before delivery were included.

Of the 89 women (23 vaginal births and 66 elective C-section births) consented and enrolled in the study, 74 delivered newborns with detectable bacterial DNA in their stool. Clinical information for this analysis was derived from medical records and a investigator administed postnatal questionnaire. Information on mode of delivery, gravidity, parity, history of urinary tract infection during pregnancy, antibiotic use during pregnancy, gestational age, birth weight, birth length, head circumference, placenta weight, sex and race was derived from medical records. Pre-pregnancy weight (kg) and height (m) were self-reported on the postnatal questionnaire. Self-reported pre-pregnancy weight has been shown to be reported to be within a few pounds of actual pre-pregnancy weight^51^.

Body mass index (BMI) was calculated as weight in kilograms divided by the square of height in mothers. We categorized pre-pregnancy BMI status as normal weight (<25 kg/m^2^), overweight (≥25–30 kg/m^2^), and obese (≥30 kg/m^2^), but because there were few obese women, we combined overweight and obesity into one category for overweight or obese (OWOB).

### Sample collection and processing

For the current study we used approximately 5 grams of the first neonatal stool (after meconium) collected from the diapers with sterile spatulas and placed in sterile tubes at 4 °C for up to 6 hours and stored at −80 °C until processing. The stool collection was scheduled every 3 hours according to the local routine care. DNA extraction was carried out using QIAamp DNA Stool mini kit (Qiagen Inc, Chatsworth, CA, USA) according to the instructions provided by the manufacturer. Following the DNA extraction (200 uL of final volume), samples were concentrated to 20 uL using 3M Na Acetate pH 5.2, quantified using Nano Drop 1000 Spectrophotometer (Thermo Scientific™, Life Technologies) and stored at −20 °C until analysis.

### Polymerase chain reaction and 16S ribosomal RNA sequencing

The variable 4 (V4) amplicon region of 16S ribosomal RNA (rRNA) gene was sequenced on the Illumina MiSeq platform (Illumina, San Diego CA, USA) at New York University with paired-end technique using the protocol modified from Caporals *et al*. (2010)^52^. The forward primer construct (5′- AAT GAT ACG GCG ACC ACC GAG ATC TAC ACT ATG GTA ATT GTG TGC CAG CMG CCG CGG TAA-3′) contained a 5′ Illumina adapter, a forward primer pad, and the 515F primer and a two-base linker sequence (‘GT’). The reverse primer (5′- CAA GCA GAA GAC GGC ATA CGA GAT NNN NNN NNN NNN AGT CAG TCA GCC GGA CTA CHV GGG TWT CTA AT – 3′) contained the 3′ Illumina adapter, a unique 12-base error-correcting Golay barcode, a reverse primer pad, a two-base linker sequence (‘CC’) and the 806R primer. The 515F/806R V4 primers amplified the ~291 base pair region of the 16S rRNA gene.

Polymerase chain reaction (PCR) was carried out in triplicate using the Bio-Rad CFX 96 thermal cycler (Bio-Rad, Hercules CA, USA). The PCR mix contained 0.2 mM forward and reverse primers, 1 ul template DNA, 10 ul 5 Prime Hot Master Mix (5 PRIME, Gaithersburg MD, USA) and 12 ul of MoBio PCR certified water (MO BIO Laboratories, Calsbad CA, USA). Thermal cycling consisted of 94 °C for 3 minutes, followed by 35 cycles of 94 °C for 45 seconds, 50 °C for 60 seconds and 72 °C for 90 seconds, with a final extension of 10 minutes at 72 °C to confirm full amplification. Replicate amplicons were pooled and the DNA concentrations were determined using the Quant-iT PicoGreen dsDNA reagent and kit (Invitrogen, Grand Island NY, USA) based on the manufacturer’s instructions. Fluorescence was measured on the Perkin-Elmer Victor Plate reader using the 490/535 nm excitation/emission filter pair with measurement time 0.1 seconds. The amplicons were then pooled in equimolar ratios and purified using QIA quick PCR purification kit (Qiagen Inc, Chatsworth, CA, USA). The final concentration of cleaned DNA amplicon was determined using the Qubit PicoGreen dsDNA HS assay kit (Invitrogen, Grand Island, NY, USA) and was sequenced using the paired-end technique. Reagents for DNA extraction and for PCR amplification were sequenced as controls^53^. There were no problems to get proper DNA quantity (>100 ng per sample) by PCR amplification.

### 16S rRNA gene sequence analysis

We processed sequence reads using the Quantitative Insights Into Microbial Ecology (QIIME) 1.8.0 software package^52^ to analyze 16S rRNA gene sequence data. The quality filtrated sequences (Phred >=Q20) were used to identify and quantify Operational Taxonomic Units (OTUs), with an open-reference OTU picking method using 97% identity to the Greengenes database (v13_8)^54^ by UCLUST^55^ and PyNAST^56^ alignment algorithms. FastTree^57^ was used to construct a phylogenetic tree. ChimeraSlayer was used to identify and remove chimeric sequences.

Samples were rarefied to 1,634 reads per sample, and evaluated for alpha diversity (microbial diversity within samples) and beta diversity (community diversity divergence between samples). Alpha rarefaction was plotted using the phylogenetic distance and the detected number of species metrics. We randomly subsampled sequences over a range of specified depths and calculated alpha diversity for each sample, with 10 independent iterations at each depth. Principle coordinate analysis (PCoA) was used to measure beta diversity on normalized OTU tables using weighted and unweighted Unifrac distance^58^. The significance of PCoA plots were analyzed using permutational multivariate analysis of variance (PERMANOVA), which uses distance metrics to confirm the strength and statistical significance of sample groupings^59^. We used 999 Monte Carlo permutations in QIIME to assess statistical significance of between-group diversity metrics.

The linear discriminant analysis (LDA) effect size (LEfSe) algorithm (found in the online interface Galaxy: https://huttenhower.sph.harvard.edu) was used to identify significant differences in relative abundance of bacterial taxonomy. For the LEfSe analysis, the α value for the factorial Kruskal-Wallis test was set at 0.05. The threshold for the logarithmic LDA score was 3.0. Taxonomic predictions in species level of OTUs detected by LEfSe were carried out using BLASTN against a 16S rRNA gene reference database from NCBI at a cut-off of 97% identity.

### In silico metagenome prediction

Phylogenetics Investigation of Communities by Reconstruction of Unobserved States (PICRUSt) was used to predict functional metagenomes from the 16S rRNA gene^18^. Kyoto Encyclopedia of Genes and Genomes (KEGG) Orthologs classification was used to classify predicted metagenomes^60^. LEfSe algorithm was used to detect significant differences of gene content between groups (LDA score > 3.0-fold).

### Analysis of clinical characteristics

Descriptive analyses were performed according to the parametric or nonparametric distribution of data, as identified by the Kolmogorov–Smirnov test. We used a Student’s *t* test for unpaired parametric, a Mann-Whitney test for unpaired nonparametric data, and a Chi-square test to compare proportions. A *p* < 0.05 was considered statistically significant.

## Additional Information

**How to cite this article**: Mueller, N. T. *et al*. Birth mode-dependent association between pre-pregnancy maternal weight status and the neonatal intestinal microbiome. *Sci. Rep.* **6**, 23133; doi: 10.1038/srep23133 (2016).

## Supplementary Material

Supplementary Information

## Figures and Tables

**Table 1:** Characteristics of mothers and neonates according to maternal pre-pregnancy BMI.

	All	Maternal Pre-pregnancy BMI	
		BMI < 25 kg/m^2^	BMI ≥ 25 kg/m^2^
Participants (n)	74	43	31
Maternal age, *years*, mean (SD)	30.7 (5.0)	30.3 (4.9)	31.2 (5.0)
Cesarean delivery, n (%)	56 (75.7)	30 (69.8)	26 (83.9)
Vaginal delivery, n (%)	48 (58)	13 (30.2)	5 (16.1)
Pregnancy weight gain, *kg*, mean (SD)	13.3 (4.8)	13.4 (4.7)	13.2 (5.1)
Girls, n (%)	36 (48.7)	23 (53.5)	13 (41.9)
Gestational age, *weeks*, mean (SD)	38.8 (0.8)	38.8 (0.9)	38.7 (0.7)
Birth weight, *grams*, mean (SD)	3257.2 (361.1)	3234.5 (332.0)	3288.6 (401.6)

**Table 2:** Predictive functional profiling of microbial communities, using 16s rRNA marker gene sequences, from fecal microbiota of neonates delivered vaginally* to normal weight vs. overweight or obese mothers.

		Vaginally Delivered Neonates	
Predicted Metagenomic Functional Capabilities		Normal Weight Mothers (n = 13)	Overweight or Obese Mothers (n = 5)
Energy Metabolism	Methane Metabolism		X
	Carbon Fixation Pathways		X
Amino Acid Metabolism	Lysine Degradation	X	
	Tryptophan Metabolism	X	
	Valine, Leucine & Isoleucine Degradation	X	
	Alanine, Aspartate & Glutamate Metabolism		X
	Histidine Metabolism		X
Carbohydrate Metabolism	Butanoate Metabolism	X	
	Amino Sugar & Nucleotide Sugar Metabolism		X
	Fructose & Mannose Metabolism		X
	Galactose Metabolism		X
	Pentose & Glucuronate Interconversions		X
Lipid Metabolism	Fatty Acid Metabolism	X	
	Sphingolipid Metabolism		X
Glycan Metabolism	Glycosaminoglycan Degradation		X
	Other Glycan Degradation		X
Other	Purine Metabolism		X
	Benzoate Metabolism	X	
	Protein Kinases	X	
	Streptomycin Biosynthesis		X

“X” denotes an over represented metabolic route in the predicted metagenome of newborn feces by pre-pregnancy maternal BMI status. LDA Effect Size (>3.0-fold) was used to detect significant differences.

^*^There were no differences in predictive functional profiling of microbial communities for neonates delivered by Cesarean section to normal weight vs. overweight or obese mothers.
