# Supplementary material for: Birth mode-dependent association between pre-pregnancy maternal weight status and the neonatal intestinal microbiome
Source: Sci Rep. 2016 Apr 1;6:23133. doi: 10.1038/srep23133 (PMC4817027; doi:10.1038/srep23133)
Supplement: Supplementary Information [file srep23133-s1.pdf]

Manuscript Title: Birth mode-dependent association between pre-pregnancy maternal weight status and the neonatal intestinal microbiome

Authors: Noel T Mueller[1,*], PhD; Hakdong Shin[2], PhD; Aline Pizoni[3]; Isabel C Werlang[3], PhD; Ursula Matte[5], PhD; Marcelo Z Goldani[4,6], MD, PhD; Helena AS Goldani[3,4,6] MD, PhD; Maria Gloria Dominguez-Bello[2*]

Affiliations:

[1]Department of Epidemiology, Johns Hopkins Bloomberg School of Public Health, Baltimore, MD
[2]New York University School of Medicine, New York, NY
[3]Post-Graduate Program Sciences in Gastroenterology and Hepatology, Faculty of Medicine, Federal University of Rio Grande do Sul, Porto Alegre – RS, Brazil.
[4]Laboratory of Translational Pediatrics, Hospital de Clínicas de Porto Alegre, Federal University of Rio Grande do Sul, Porto Alegre – RS, Brazil
[5]Department of Genetics, Federal University of Rio Grande do Sul, Porto Alegre – RS, Brazil
[6]Department of Pediatrics, Faculty of Medicine, Federal University of Rio Grande do Sul, Porto Alegre – RS, Brazil

Corresponding authors:

Noel Theodore Mueller
Address: 2024 E. Monument St., Suite 2-600, Baltimore, MD, 21287
Email: nmuelle4@jhu.edu
Phone: 410-614-7628

Maria Gloria Dominguez-Bello
Address: 462 First Avenue, New York, NY 10016
Email: Maria.Dominguez-Bello@nyumc.org
Phone: 646-501-0703

SUPPLEMENTARY FILES

Legends:

Table S1. Summary of analyzed 16S rRNA sequence data.

Table S2. Comparing the relative abundance of *Bacteroides* operational taxonomic units in the stool of neonates using BLASTN.

Figure S1. Bacterial beta diversity in newborn feces according to mode of delivery. Weighted (left) and unweighted (right) UniFrac distances were used to evaluate beta diversity. PERMANOVA was used to test dissimilarity.

Figure S2. Phylogenetic diversity (**A**), observed species (**B**), and species richness (**C**) plots for bacterial operating taxonomic units (OTUs) in newborn feces according to mode of delivery. All communities were rarefied up to 1,634 reads per sample to calculate bacterial diversity (means ± 1 SE).

Figure S3. Phylogenetic diversity (**A**), observed species (**B**), and species richness (**C**) plots for bacterial operating taxomonic units (OTUs) in neonatal feces according to mode of delivery and maternal pre-pregnancy BMI. All communities were rarefied up to 1,634 reads per sample to calculate bacterial diversity (means ± 1 SE).

Figure S4. Differences in relative abundance of bacterial taxa in newborn feces according to mode of delivery. **A**. Bacterial taxonomic plots are shown at the phylum and genus levels. Each taxon representing >1% of the average relative abundance in study groups is indicated by a different color. Taxa are reported at the lowest identifiable level, indicated by the letter preceding the underscore: f, family; g, genus. **B**. Histogram of significant bacterial taxonomic biomarkers in each group; LDA Effect Size (>3.0-fold) was used to determine significant biomarkers.

Figure S5. Differences in relative abundance of bacterial taxa in newborn feces by pre-pregnancy maternal BMI according to delivery mode. Bacterial taxonomic plots at the phylum (**A**) and genus (**B**) levels for each newborn. Each taxon representing >1% of the average relative abundance is indicated by a different color. Taxa are reported at the lowest identifiable level, indicated by the letter preceding the underscore: f, family; g, genus.

Figure S6. Bacterial beta diversity in the feces of Cesarean (C-section) delivered newborns according to whether their (**A**) mothers were treated or were not treated with prenatal antibiotics, or whether (**B**) they were or were not breastfed in first 24 hours after birth. Weighted UniFrac distances were used to evaluate diversity and PERMANOVA was used to test dissimilarity.

Table S1. Summary of analyzed 16S rRNA sequence data.

	**Cesarean delivered neonates (n = 56)**		**Vaginally delivered neonates (n = 18)**		
	Maternal pre-pregnancy BMI status				
**Characteristics**	**Normal weight**	**Overweight or Obese**	**Normal weight**	**Overweight or Obese**	**Total**
Number of samples	30	26	13	5	74
Total number of sequences	261,408	266,333	144,133	53,215	725,089
Mean number of sequences (± SEM)	8,714 ± 662	10,244 ± 863	11,087 ± 926	10,643 ± 2,209	9,799 ± 474
Total number of sequences yielding OTUs	250,004	258,927	125,657	47,326	681,914 (6.0% loss)
Mean number of sequences yielding OTUs (± SEM)	8,333 ± 639	9,959 ± 832	9,666 ± 1,018	9,465 ± 2,453	9215 ± 468
Number of observed OTU types	2,353	1,822	1,219	531	3,717

Table S2. Comparing the relative abundance of *Bacteroides* operational taxonomic units in the stool of neonates using BLASTN.

Differences, according to maternal pre-pregnancy BMI, in the (mean) relative abundance of operational taxonomic units (OTU) within the *Bacteroides* genus (g) among stools from neonates **delivered vaginally.**								
					Maternal BMI <25 kg/m^2	Maternal BMI ≥25 kg/m^2		
Predicted taxonomy	OTU ID	Query length (nt)	Best hit (BLASTN)	Identities	Mean	Mean	Test Statistic	P value
g__Bacteroides;s__caccae	196664	204	Bacteroides caccae strain JCM 9498	100%	2.92	3.60	4.40	0.04
g__Bacteroides	1992	253	Bacteroides caccae strain JCM 9498	99%	0.00	4.80	5.51	0.02
g__Bacteroides	161423	253	Bacteroides dorei strain 175	98%	22.85	127.80	8.83	<0.01
g__Bacteroides	172777	253	Bacteroides dorei strain 175	98%	4.62	12.00	7.19	0.01
g__Bacteroides	177222	253	Bacteroides dorei strain 175	98%	1.77	2.60	5.32	0.02
g__Bacteroides	4447950	253	Bacteroides stercoris strain JCM 9496	100%	0.00	4.80	5.51	0.02
g__Bacteroides	2875735	198	Bacteroides vulgatus strain ATCC 8482	100%	14.15	156.60	9.26	<0.01
g__Bacteroides	3426658	205	Bacteroides vulgatus strain ATCC 8482	100%	15.69	252.40	9.26	<0.01
g__Bacteroides	3439403	213	Bacteroides vulgatus strain ATCC 8482	100%	6.62	84.80	9.51	<0.01
g__Bacteroides	3563235	219	Bacteroides vulgatus strain ATCC 8482	100%	19.54	263.40	9.26	<0.01
g__Bacteroides	3600504	253	Bacteroides vulgatus strain ATCC 8482	100%	215.38	3212.60	8.06	<0.01
g__Bacteroides	157327	253	Bacteroides vulgatus strain ATCC 8482	99%	0.46	8.80	12.84	<0.01
g__Bacteroides	182255	253	Bacteroides vulgatus strain ATCC 8482	99%	0.62	4.00	8.56	<0.01
g__Bacteroides	183662	246	Bacteroides vulgatus strain ATCC 8482	99%	0.31	2.20	5.35	0.02
g__Bacteroides	184209	253	Bacteroides vulgatus strain ATCC 8482	99%	0.31	3.00	8.61	<0.01
g__Bacteroides	211706	253	Bacteroides vulgatus strain ATCC 8482	99%	0.15	3.20	5.00	0.03
g__Bacteroides	844375	207	Bacteroides vulgatus strain ATCC 8482	99%	0.23	28.20	8.59	<0.01
g__Bacteroides	1508541	253	Bacteroides vulgatus strain ATCC 8482	99%	0.69	9.60	6.37	0.01

g__Bacteroides	1749079	192	Bacteroides vulgatus strain ATCC 8482	99%	1.00	20.80	10.59	<0.01
g__Bacteroides	1919007	253	Bacteroides vulgatus strain ATCC 8482	99%	0.00	2.60	5.51	0.02
g__Bacteroides	2134452	198	Bacteroides vulgatus strain ATCC 8482	99%	1.92	35.00	9.56	<0.01
g__Bacteroides	2137001	201	Bacteroides vulgatus strain ATCC 8482	99%	5.15	94.80	10.19	<0.01
g__Bacteroides	3256931	219	Bacteroides vulgatus strain ATCC 8482	99%	1.77	25.00	9.86	<0.01
g__Bacteroides	3272632	205	Bacteroides vulgatus strain ATCC 8482	99%	4.69	69.80	7.81	0.01
g__Bacteroides	3588390	253	Bacteroides vulgatus strain ATCC 8482	99%	38.69	545.20	8.77	<0.01
g__Bacteroides	3887769	253	Bacteroides vulgatus strain ATCC 8482	99%	0.23	2.00	7.94	<0.01
g__Bacteroides	4232045	253	Bacteroides vulgatus strain ATCC 8482	99%	2.92	165.60	4.46	0.03
g__Bacteroides	4357811	253	Bacteroides vulgatus strain ATCC 8482	99%	0.62	2.60	5.52	0.02
g__Bacteroides	4468234	253	Bacteroides vulgatus strain ATCC 8482	99%	39.54	396.40	6.55	0.01
g__Bacteroides	270094	253	Bacteroides vulgatus strain ATCC 8482	98%	0.69	22.20	4.24	0.04
g__Bacteroides	4256470	253	Bacteroides xylanisolvens strain XB1A	100%	0.00	9.40	5.51	0.02
Differences, according to maternal pre-pregnancy BMI, in the (mean) relative abundance of operational taxonomic units (OTU) within the *Bacteroides* genus (g) among stools from neonates **delivered by C-section.**								
					Maternal BMI <25 kg/m^2	Maternal BMI $\geq$25 kg/m^2		
Predicted taxonomy	OTU ID	Query length (nt)	Best hit (BLASTN)	Identities	Mean	Mean	Test Statistic	P value
g__Bacteroides	3563235	219	Bacteroides vulgatus strain ATCC 8482	100%	3.53	0.00	4.66	0.03

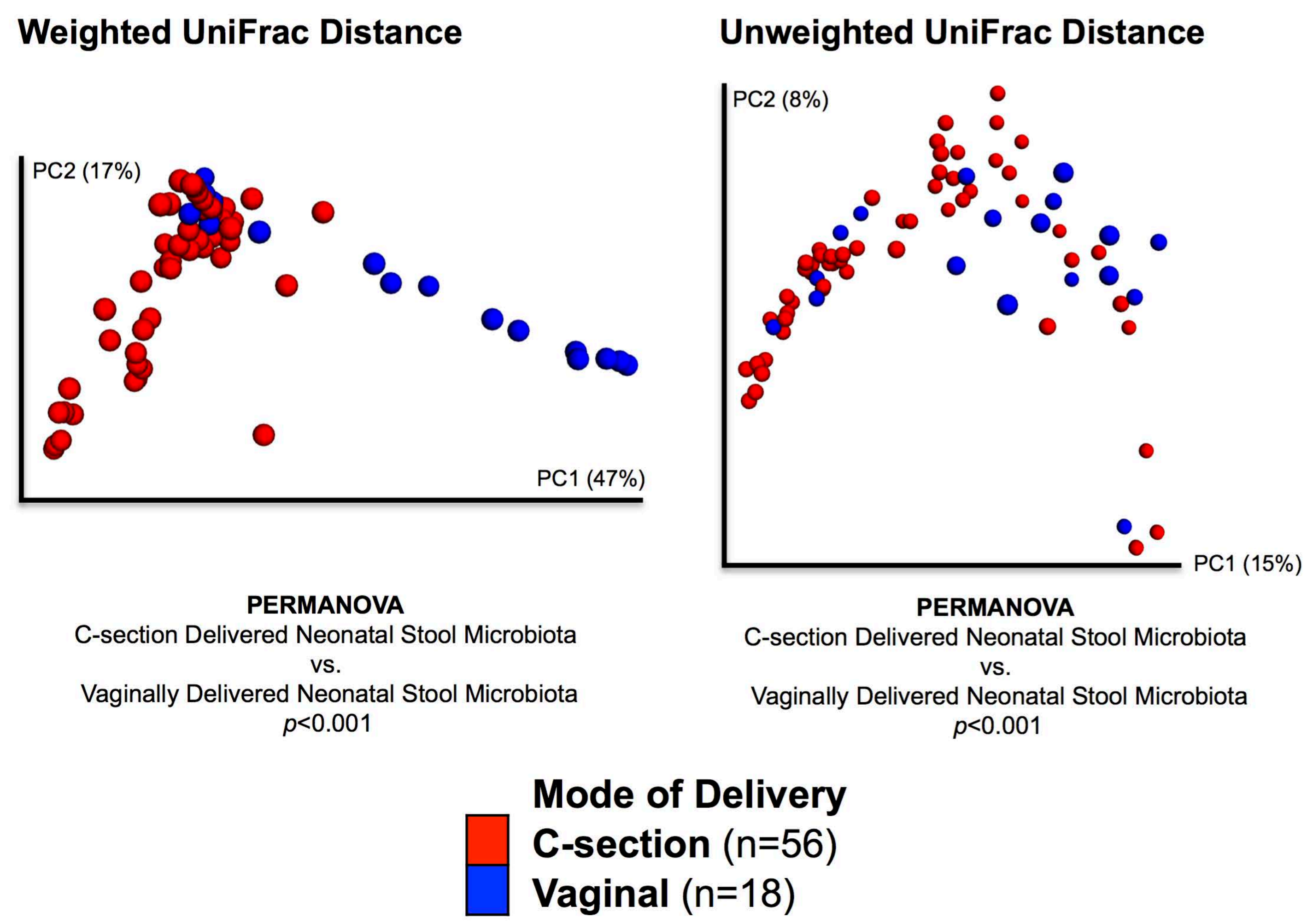
Weighted UniFrac Distance
PC2 (17%)
PC1 (47%)
PERMANOVA
C-section Delivered Neonatal Stool Microbiota
vs.
Vaginally Delivered Neonatal Stool Microbiota
p<0.001
Unweighted UniFrac Distance
PC2 (8%)
PC1 (15%)
PERMANOVA
C-section Delivered Neonatal Stool Microbiota
vs.
Vaginally Delivered Neonatal Stool Microbiota
p<0.001
Mode of Delivery
C-section (n=56)
Vaginal (n=18)

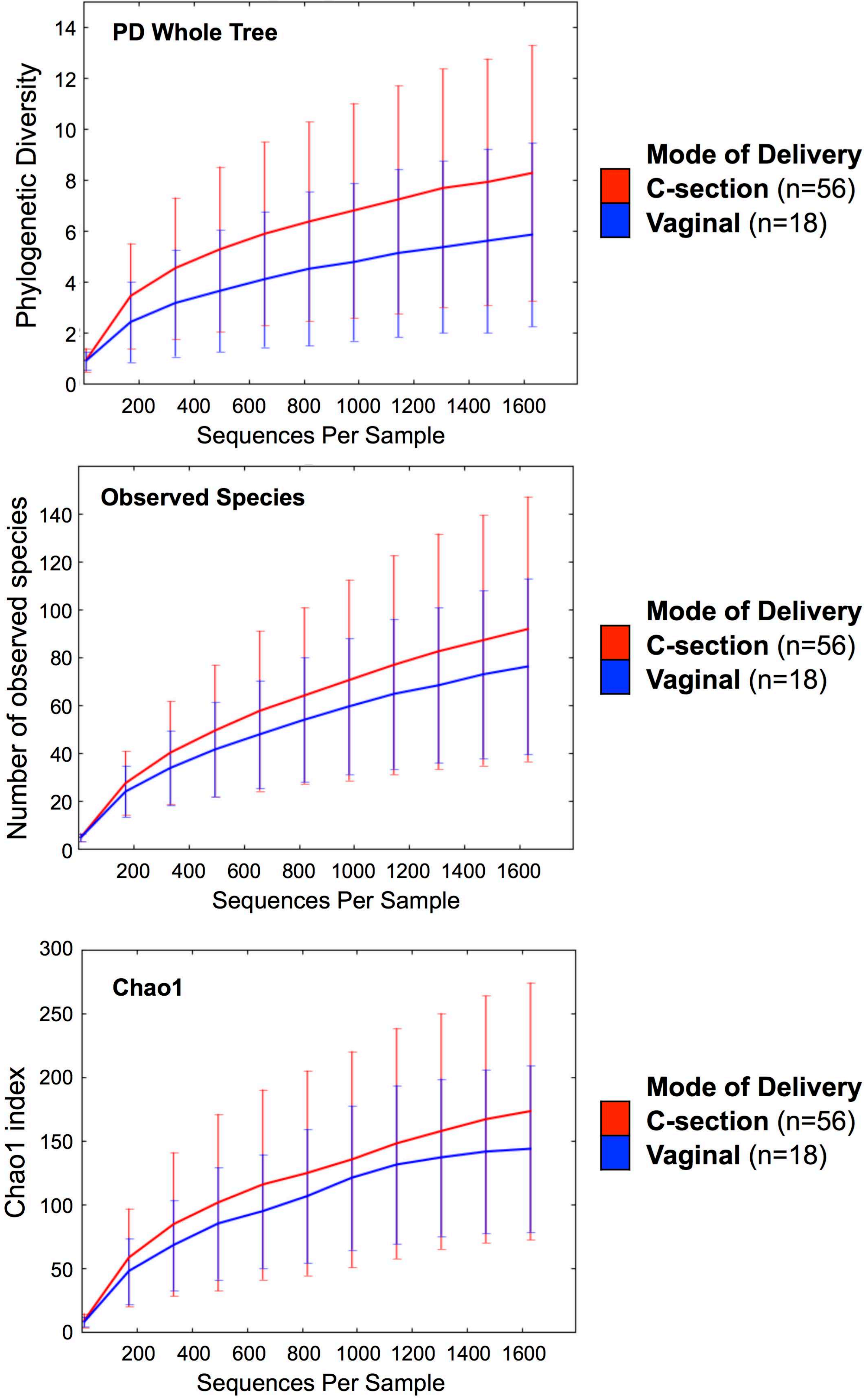

PD Whole Tree
Phylogenetic Diversity
Sequences Per Sample
Mode of Delivery
C-section (n=56)
Vaginal (n=18)
Observed Species
Number of observed species
Sequences Per Sample
Mode of Delivery
C-section (n=56)
Vaginal (n=18)
Chao1
Chao1 index
Sequences Per Sample
Mode of Delivery
C-section (n=56)
Vaginal (n=18)

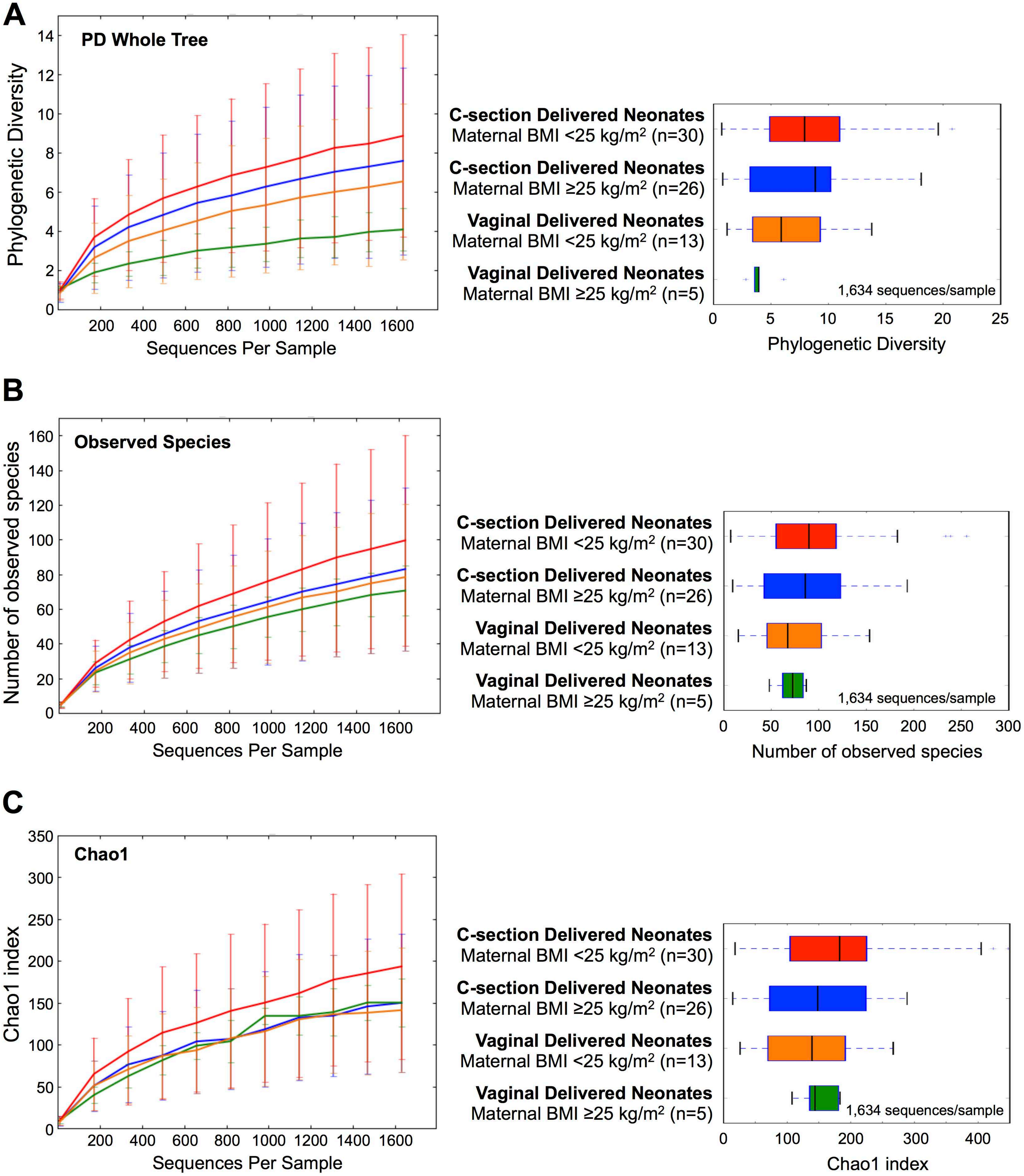
A
PD Whole Tree
Phylogenetic Diversity
Sequences Per Sample
C-section Delivered Neonates
Maternal BMI <25 kg/m2 (n=30)
C-section Delivered Neonates
Maternal BMI ≥25 kg/m2 (n=26)
Vaginal Delivered Neonates
Maternal BMI <25 kg/m2 (n=13)
Vaginal Delivered Neonates
Maternal BMI ≥25 kg/m2 (n=5)
1,634 sequences/sample
Phylogenetic Diversity
B
Observed Species
Number of observed species
Sequences Per Sample
C-section Delivered Neonates
Maternal BMI <25 kg/m2 (n=30)
C-section Delivered Neonates
Maternal BMI ≥25 kg/m2 (n=26)
Vaginal Delivered Neonates
Maternal BMI <25 kg/m2 (n=13)
Vaginal Delivered Neonates
Maternal BMI ≥25 kg/m2 (n=5)
1,634 sequences/sample
Number of observed species
C
Chao1
Chao1 index
Sequences Per Sample
C-section Delivered Neonates
Maternal BMI <25 kg/m2 (n=30)
C-section Delivered Neonates
Maternal BMI ≥25 kg/m2 (n=26)
Vaginal Delivered Neonates
Maternal BMI <25 kg/m2 (n=13)
Vaginal Delivered Neonates
Maternal BMI ≥25 kg/m2 (n=5)
1,634 sequences/sample
Chao1 index

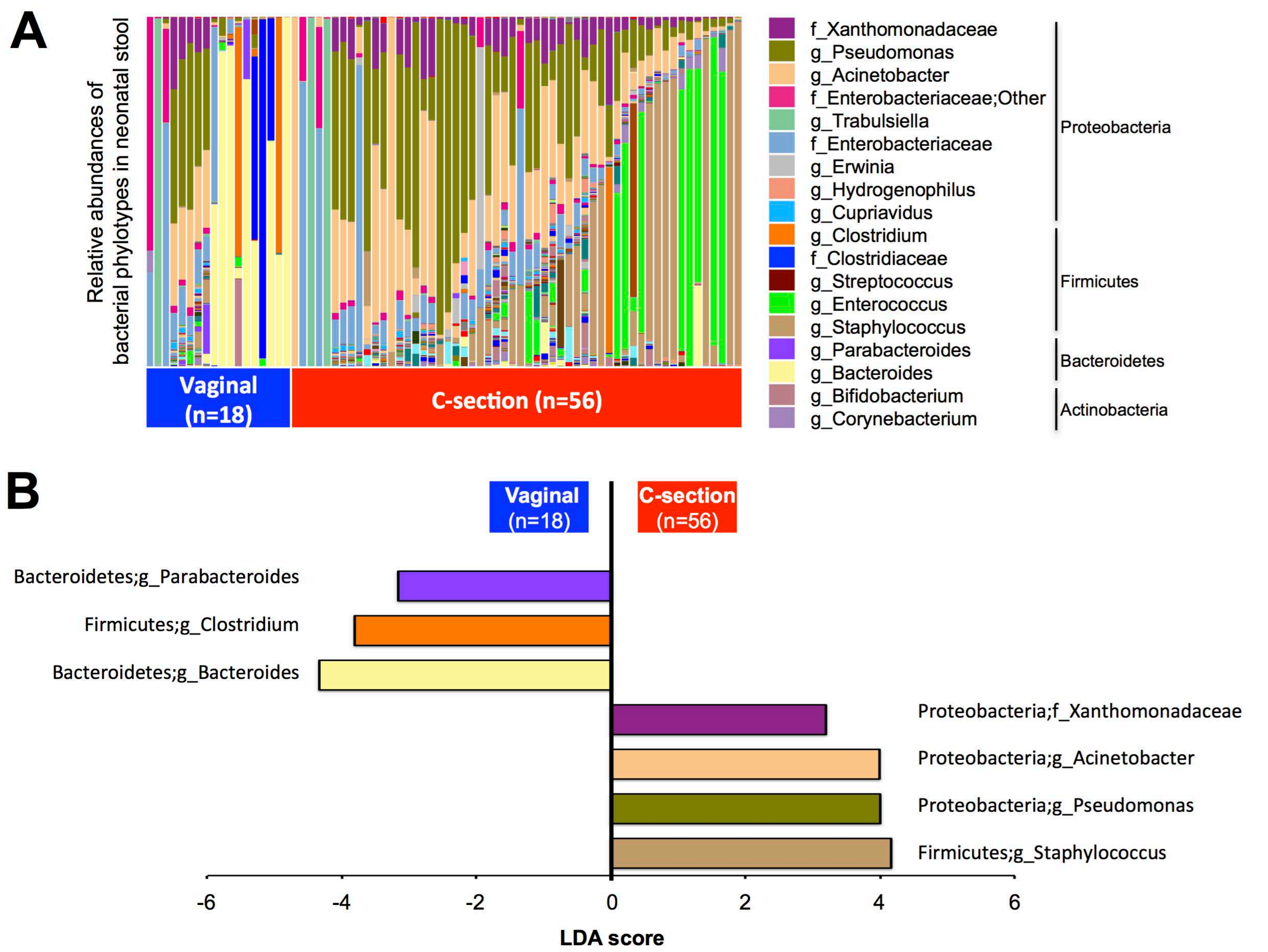

A
Relative abundances of bacterial phylotypes in neonatal stool
Vaginal (n=18)
C-section (n=56)
f_Xanthomonadaceae
g_Pseudomonas
g_Acinetobacter
f_Enterobacteriaceae;Other
g_Trabulsiella
f_Enterobacteriaceae
g_Erwinia
g_Hydrogenophilus
g_Cupriavidus
g_Clostridium
f_Clostridiaceae
g_Streptococcus
g_Enterococcus
g_Staphylococcus
g_Parabacteroides
g_Bacteroides
g_Bifidobacterium
g_Corynebacterium
Proteobacteria
Firmicutes
Bacteroidetes
Actinobacteria
B
Vaginal (n=18)
C-section (n=56)
Bacteroidetes;g_Parabacteroides
Firmicutes;g_Clostridium
Bacteroidetes;g_Bacteroides
Proteobacteria;f_Xanthomonadaceae
Proteobacteria;g_Acinetobacter
Proteobacteria;g_Pseudomonas
Firmicutes;g_Staphylococcus
-6
-4
-2
0
2
4
6
LDA score

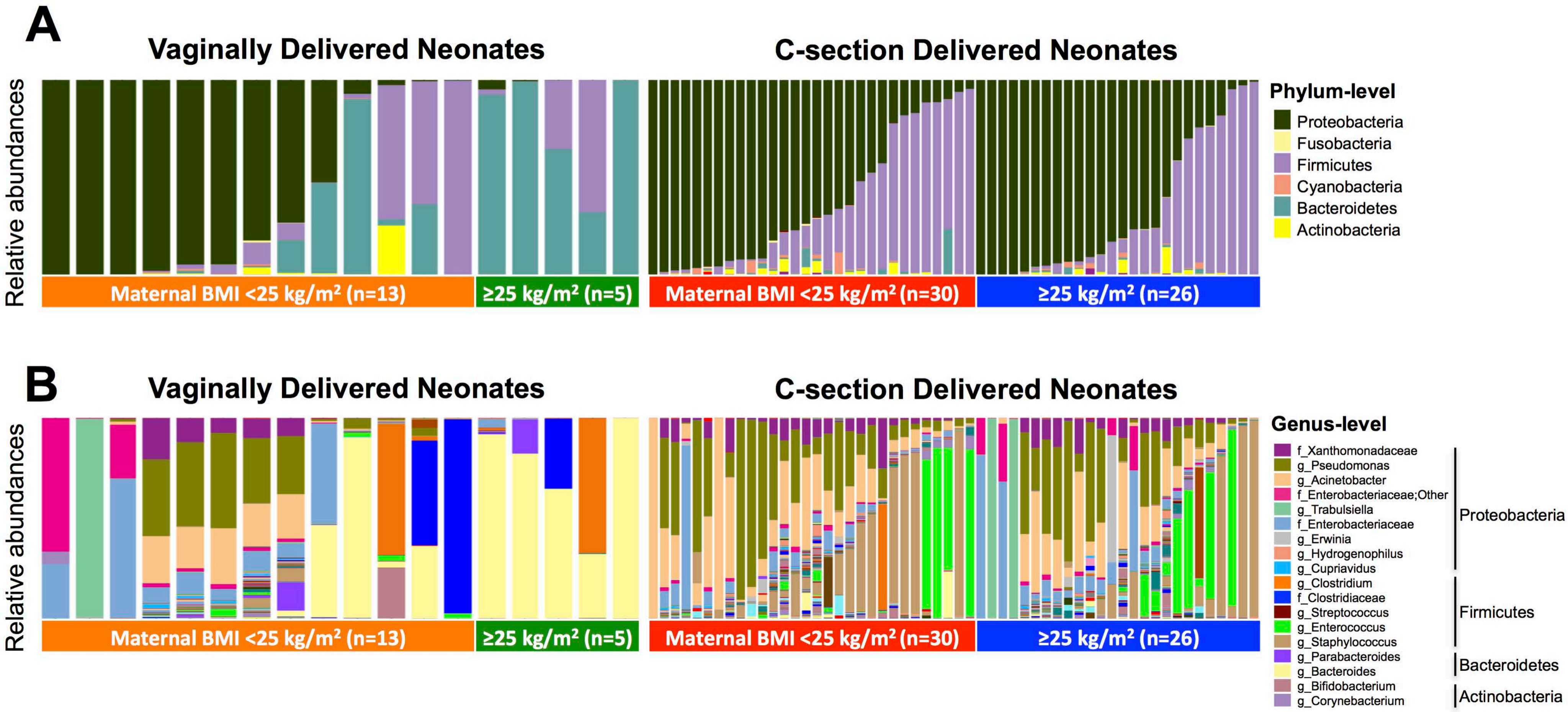
A
Vaginally Delivered Neonates
C-section Delivered Neonates
Relative abundances
Phylum-level
Proteobacteria
Fusobacteria
Firmicutes
Cyanobacteria
Bacteroidetes
Actinobacteria
Maternal BMI <25 kg/m² (n=13)
≥25 kg/m² (n=5)
Maternal BMI <25 kg/m² (n=30)
≥25 kg/m² (n=26)
B
Vaginally Delivered Neonates
C-section Delivered Neonates
Relative abundances
Genus-level
f_Xanthomonadaceae
g_Pseudomonas
g_Acinetobacter
f_Enterobacteriaceae;Other
g_Trabulsiella
f_Enterobacteriaceae
g_Erwinia
g_Hydrogenophilus
g_Cupriavidus
g_Clostridium
f_Clostridiaceae
g_Streptococcus
g_Enterococcus
g_Staphylococcus
g_Parabacteroides
g_Bacteroides
g_Bifidobacterium
g_Corynebacterium
Proteobacteria
Firmicutes
Bacteroidetes
Actinobacteria
Maternal BMI <25 kg/m² (n=13)
≥25 kg/m² (n=5)
Maternal BMI <25 kg/m² (n=30)
≥25 kg/m² (n=26)

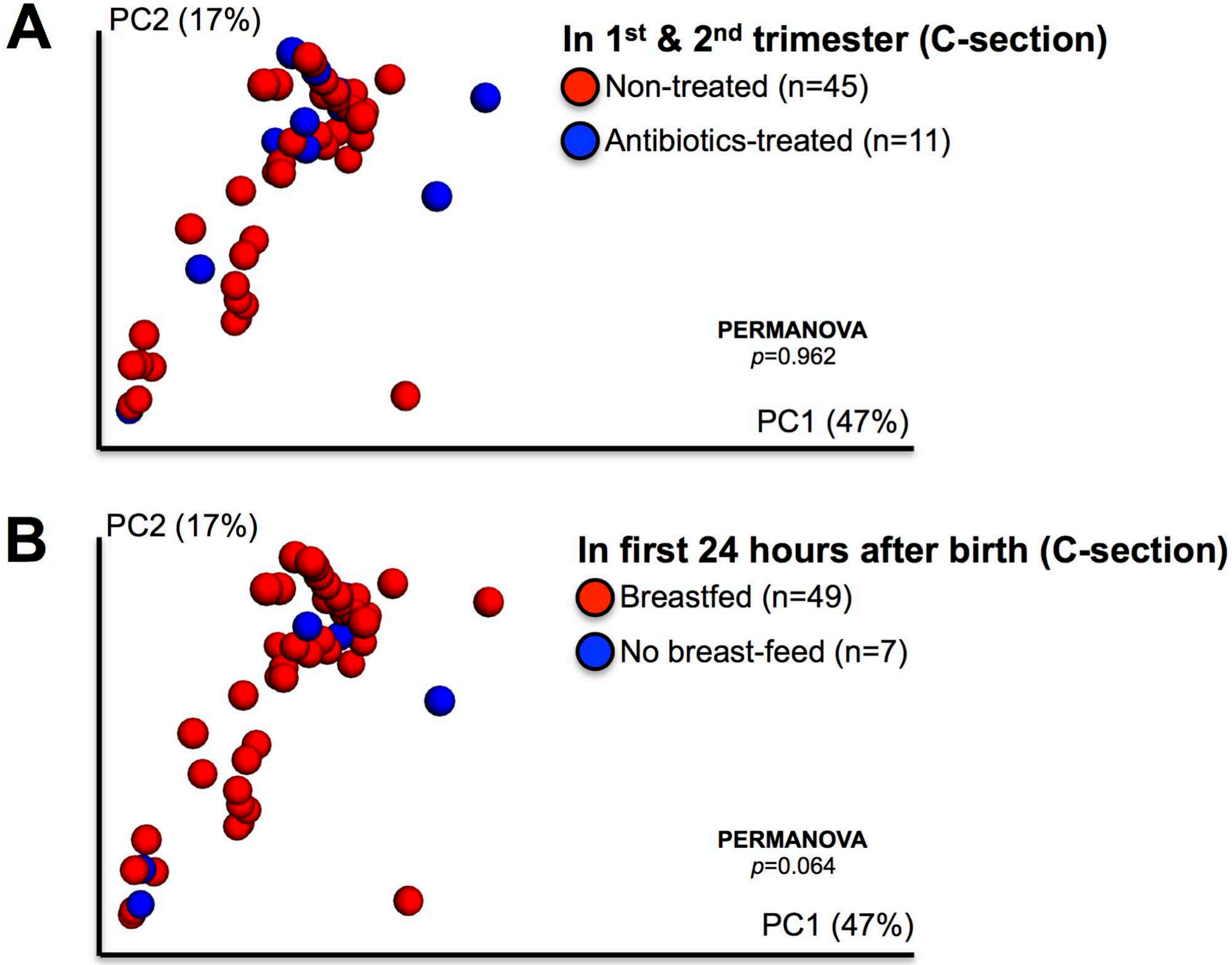
A
PC2 (17%)
In 1st & 2nd trimester (C-section)
Non-treated (n=45)
Antibiotics-treated (n=11)
PERMANOVA
p=0.962
PC1 (47%)
B
PC2 (17%)
In first 24 hours after birth (C-section)
Breastfed (n=49)
No breast-feed (n=7)
PERMANOVA
p=0.064
PC1 (47%)
